# Comparison of two invitation-based methods for human papillomavirus (HPV) self-sampling with usual care among un- and under-screened Māori, Pacific and Asian women: study protocol for a randomised controlled community trial to examine the effect of self-sampling on participation in cervical-cancer screening

**DOI:** 10.1186/s12885-019-6401-y

**Published:** 2019-12-09

**Authors:** Naomi Brewer, Karen Bartholomew, Anna Maxwell, Jane Grant, Helen Wihongi, Collette Bromhead, Nina Scott, Sue Crengle, Chris Cunningham, Jeroen Douwes, John D. Potter

**Affiliations:** 1 Centre for Public Health Research, College of Health, Massey University, PO Box 756, Wellington, 6140 New Zealand; 2 Waitematā District Health Board (DHB) and Auckland DHB, Private Bag 93-503, Takapuna, Auckland, 0740 New Zealand; 3 School of Health Sciences, Massey University, Wellington, New Zealand; 4 University of Auckland, Waikato District Health Board, Hamilton, New Zealand; 5 Department of Preventive and Social Medicine, University of Otago, Dunedin, New Zealand; 6 Research Centre for Māori Health and Development, Massey University, Wellington, New Zealand; 7 Centre for Public Health Research, Massey University, Wellington, New Zealand

**Keywords:** HPV DNA testing, Home-based, Clinic-based, Self-sample, Cervical screening, Participation

## Abstract

**Background:**

Māori, Pacific and Asian women in New Zealand have lower cervical-cancer screening rates than European women, and there are persistent inequities in cervical cancer outcomes for Māori and Pacific women. Innovative ways to address access barriers are required. New Zealand is transitioning to screening with human papillomavirus (HPV) DNA testing, which could allow women themselves, rather than a clinician, to take the sample. Internationally, self-sampling has been found to increase screening participation rates. The aim of this open-label community-based randomised controlled trial is to investigate whether self-sampling increases screening participation among un- and under-screened Māori, Pacific and Asian women in New Zealand.

**Methods/design:**

We aim to invite at least 3550 un- or under-screened (≥5 years overdue) Māori, Pacific and Asian women (1050, 1250, 1250 respectively), aged 30–69 years, for screening. The three study arms are: usual care in which women are invited to attend a clinic for a standard clinician-collected cytology test; clinic-based self-sampling in which women are invited to take a self-sample at their usual general practice; and mail-out self-sampling in which women are mailed a kit and invited to take a self-sample at home. Women will be randomised 3:3:1 to the clinic and mail-out self-sampling groups, and usual care. There is also a nested sub-study in which non-responding women in all allocation groups, when they subsequently present to the clinic for other reasons, are offered clinic or home-kit self-sampling. The primary outcome will be the proportion of women who participate (by taking a self-sample or cytology test).

**Discussion:**

This trial is the first to evaluate the effectiveness of mailed self-sampling in New Zealand and will be one of the first internationally to evaluate the effectiveness of opportunistic in-clinic invitations for self-sampling. The trial will provide robust evidence on the impact on participation proportions from different invitation approaches for HPV self-sampling in New Zealand un- and under-screened Māori, Pacific and Asian women.

**Trial registration:**

ANZCTR Identifier: ACTRN12618000367246 (date registered 12/3/2018) https://www.anzctr.org.au/Trial/Registration/TrialReview.aspx?id=371741&isReview=true; UTN: U1111–1189-0531.

## Background

The New Zealand National Cervical Screening Programme (NCSP) has been established for 28 years. Although cervical cancer incidence has declined in both Māori (the Indigenous people of New Zealand) and non-Māori, invasive disease persists, predominantly in women who are not screened or who are under-screened [1, 2]. In 2014, cervical cancer registration (New Zealand Cancer Registry) was twice as high, and mortality rates three times as high in Māori women compared to non-Māori women [3]. Pacific women in New Zealand also have higher cervical cancer incidence [4].

Approximately 75% of cervical cancer cases among Pacific women in New Zealand have been found to occur in women who have not attended cervical screening [5]. Similarly, around 70% of Asian women, and 59% of Māori women diagnosed with cervical cancer were shown to have not been screened [5]. Cervical-cancer screening is recommended every 3 years in New Zealand. In June 2019, the three-year coverage proportion for 25–69 year old women was 60.9% for Asians, 66.6% for Pacific, 66.8% for Māori, and 75.6% for others (mostly European) [6].

There are many reasons for low participation in screening, which can be summarised as health system failure, attitudinal bias (racism), differential access, and quality of care [1, 2, 7–9]. Actions to reduce these barriers, including no-cost targeted testing (‘free smears’) and tailored practice-level data-matching to identify under-screened women to offer support, have been undertaken across the country. However, despite these measures, there has been little change in participation among Māori, Pacific and Asian women in the last decade [6]. Novel strategies are therefore required to change the landscape of cervical screening.

New Zealand is currently assessing policy options to inform a transition from traditional cervical screening by cytology (previously a ‘pap smear’, now liquid-based cytology (LBC)) to a human papillomavirus (HPV)-based programme. Because persistent cervical infection with oncogenic HPV causes virtually all cervical cancers, [10, 11] the World Health Organization [12] recommends primary HPV screening for early detection of cervical cancer. In high-resource settings, HPV testing for primary cervical cancer screening could: increase the efficiency of the existing programme; more effectively identify women at risk of precancerous changes; and, therefore, reduce incidence and mortality from cervical cancer [11]. In New Zealand, HPV testing of a clinician-collected sample for primary cervical cancer screening is predicted to give a 12–16% reduction in cervical cancer incidence and mortality [13].

New Zealand has an established HPV vaccination programme, now using Gardasil® 9, protecting young people of both sexes (aged 9 to 26 years) against HPV types 6, 11, 16, 18, 31, 33, 45, 52, and 58 [14]. However, in 2017, a large proportion (33%) of young women were not vaccinated [15] and the earlier vaccine (Gardasil 4; used from 2008 to 2017), in particular, does not protect against all oncogenic HPV types.

Unlike cytology assessment, HPV testing is based on viral deoxyribonucleic acid (DNA) and does not require intact cells. Therefore, a less invasive method of sampling, aimed at simplifying the screening process and reducing barriers to programme participation, may be used, such as vaginal self-sampling [16]. Self-sampling approaches have consistently shown an improved participation rate in cervical screening, including among the least well-served women who are unscreened [16–21]. The iPAP trial in Australia, in which HPV self-sampling kits were mailed to under-screened women, demonstrated 20% uptake compared with 6% for usual care [19]. HPV self-sampling has been included in the 2017 renewal of the Australian screening programme [22]. It has also been incorporated into the cervical screening programme in the Netherlands and has recently been introduced in the Capital Region of Denmark [23, 24]. In New Zealand, vaginal self-sampling is already used to test for sexually transmitted diseases [25] and has previously been suggested to be preferred by Māori women [26].

The accuracy of self-sampling in detecting high-grade precancerous cervical changes (cervical intraepithelial neoplasia grade two or higher; CIN2+) has been consistently shown to be similar to clinician-collected samples when tested for oncogenic HPVs using polymerase chain reaction (PCR) [18, 23, 27–29]. Most studies did not compare different sampling devices; however, no statistically significant difference was reported between a brush-based and a lavage-based device for CIN2+ and CIN3+ detection rates (when using a high-performing PCR DNA assay) and user comfort [30]. An inexpensive and low-tech device, the dry flocked swab, had the same accuracy for clinician-taken samples, when used with a dry tube (containing no preservative solution), as a cyto-broom used with a wet tube (containing PreservCyt® solution) in Australia [31].

Internationally, HPV self-sampling has been used with a range of invitation approaches, including in general practice clinics, [32] community-health-worker delivery [17] and by mail [31, 33]. A meta-analysis confirmed that: a range of delivery approaches are acceptable to under-screened women; there is improved participation among under-screened women; and the approach should be tailored to local populations [34].

The use of a novel HPV self-sampling technology in un- or under-screened Māori, Pacific and Asian women may improve participation as suggested by recent work in New Zealand [35, 36] and thus partially address the burden of cervical cancer in these populations. However, as recommended by Arbyn et al [27], a local trial is needed to assess feasibility, effectiveness, and cost-effectiveness before the introduction of a programme that includes self-sampling; this is the primary aim of the current study. In particular, we aim to evaluate the acceptability (assessed by participation (uptake)) of HPV self-sampling in the high-priority populations of un- and under-screened Māori, Pacific, and Asian women, with the ultimate goal of providing robust evidence for New Zealand policy decisions on cervical screening.

### Study objectives

The primary study objectives are to determine: 1) the self-sampling participation proportion in Māori, Pacific and Asian women; 2) the follow-up proportion for oncogenic HPV-positive women, and; 3) the prevalence of oncogenic HPV positivity (including genotype) and the associated colposcopic findings.

The secondary objectives are to: 1) determine the level of support needed to achieve at least 90% follow-up of oncogenic HPV-positive women to attend a primary-care smear-taker or colposcopy; 2) provide policy relevant findings for New Zealand to facilitate future implementation of national self-sampling within the next 5 years; 3) determine some acceptability issues, including: what preferences women have for invitation, sample return, and follow-up methods; whether the level of information in the printed material is appropriate and acceptable to Māori, Pacific, and Asian women; and whether further localisation or refinement is required; and 4) ultimately improve equitable health outcomes for Māori, Asian, and Pacific women in New Zealand.

## Methods/design

**Fig. 1:**
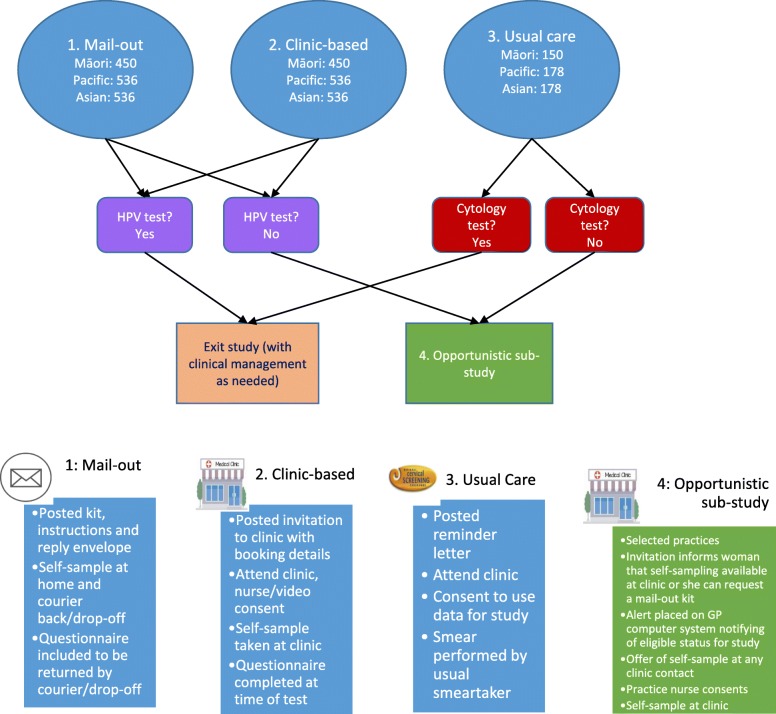
Study design overview

This is an open-label, three-arm, community-based, randomised controlled trial, with a nested sub-study (Fig. 1). We will invite a minimum of 3550 un- or under-screened (no screening recorded for the last 5 years, in accordance with the NCSP guidelines [37]) women. This group of women are least served by, and not engaged in, the current screening programme. The women will be identified through a routinely available national data-match process between Primary Health Organisations (PHOs; organisations responsible for primary care) and the NCSP, where the screening status of enrolled women is updated monthly. Participants will be Māori, Pacific or Asian women (1050, 1250, 1250 respectively), as identified by the PHO enrolment register, invited through the women’s usual primary care provider (in partnership with the research team) in the Auckland area (Waitematā District Health Board (DHB) and Auckland DHB). Ethnicity in New Zealand is self-identified as part of PHO enrolment; women identifying with multiple ethnicities are prioritised according to the New Zealand ethnicity standard for the health sector [38].

Women will be individually randomised 3:3:1 to the study arms: clinic-based self-sampling in which women are invited to take a self-sample at their usual general practice (GP); mail-out self-sampling in which women are mailed a kit and invited to take a self-sample at home; and usual care in which women are invited to attend a clinic for a standard cytology sample (attendance assessed through the NCSP-Register and GP records). We aim to randomise approximately equal numbers of women from each ethnic group to each of the study arms. There is also a nested “opportunistic” sub-study at most clinics, where women who have not responded within 3 months to their study invitation are offered, at any subsequent visit to their clinic (as suggested in Arbyn et al [27] and Lim and Sasieni [39]), either self-sampling in the clinic or the option of having a sampling kit mailed to their home. This sub-study will enable us to describe policy-relevant findings including those that inform the practical implementation of an opportunistic offer of HPV self-sampling in a clinic situation.

An overview of the schedule of enrolment, interventions, and assessments is provided in Table 1.

**Table 1:**
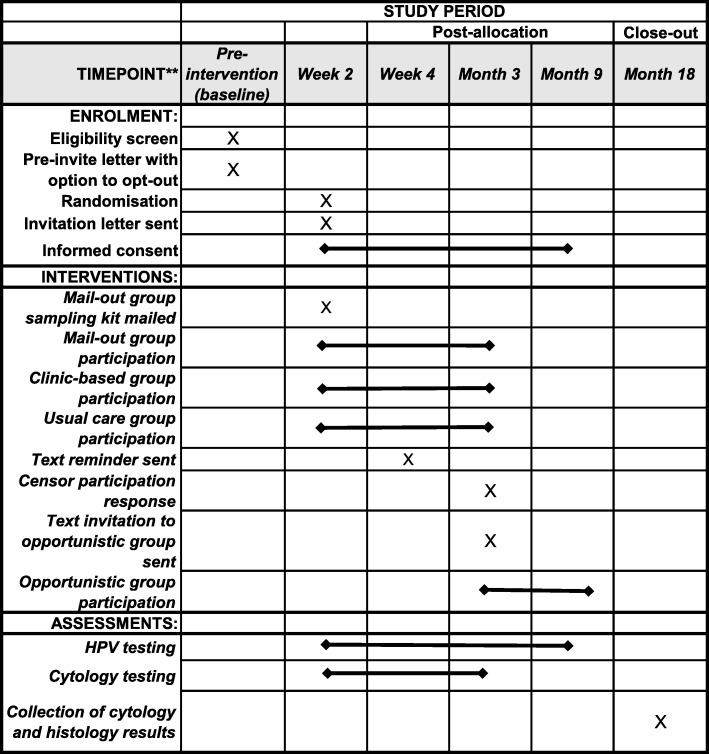
Overview of the schedule of enrolment, interventions, and assessments. Participation means providing a sample and optionally completing the questionnaire

### Exclusions

Exclusions as per the NCSP guidelines [37] will include women who: 1) have had a benign total hysterectomy; 2) have previously had or currently have cervical cancer; or 3) are symptomatic (abnormal bleeding, pelvic pain, or symptoms of a sexually transmitted infection; these women are referred for appropriate care). In addition, women will be excluded if they: 1) have previously had a high-grade lesion and have not attended for colposcopy (remaining at high clinical risk); 2) have previously had a high-grade lesion and have not completed follow-up according to NCSP guidelines [37]; 3) are currently pregnant; 4) are not eligible for New Zealand health services; 5) have not attended the specific clinic in the last 3 years; and 6) other clinical reason (e.g. terminal illness).

### Interventions

#### Invitation

Although cervical screening is recommended for all NZ women from age 20–69 [37] (the NCSP is increasing the screening start age to 25 years in 2019 [40]), the prevalence of HPV infections in women < 30 years is high and most infections clear without causing cervical abnormalities; this reduces the specificity of HPV testing [37, 41]. The age range for our study is therefore 30–69 years: to minimise unnecessary colposcopy procedures in younger women; to avoid self-sampling being the first contact for cervical screening among women who might otherwise choose current screening programme; and to maintain the specificity of the HPV testing.

All women who meet the study inclusion criteria will be sent a tailored pre-invite letter briefly explaining the study and informing them that, unless they request not to receive an invitation, they will soon be sent: 1) an invitation for cytology (usual care); 2) a self-sampling kit (mail-out); or 3) an invitation to take a self-sample at their GP clinic (clinic-based). Two weeks after the pre-invite letters have been sent, women will be randomised (excluding any who have indicated that they do not wish to receive an invitation), using a computer-generated sequence, to one of the study arms and sent an invitation.

#### Consent and sampling kit

Participating women in the usual-care arm of the study who attend will be consented by their usual smear-taker and a cytology sample taken.

Participating women in the self-sampling arms of the study will be asked to take a low-vaginal sample with a FLOQSwab™ (Copan Italia, Brescia, Italy; similar to those now widely used for self-sampling for other sexually transmitted infections) and to put the swab into a 12 mL dry tube (Sarstedt AG & Co. KG, Germany).

The clinic-based self-sampling group will receive an invitation letter and participant information brochure via their primary-care provider. The women will also be directed to a webpage [42] with translated study documents and study video clips (with subtitles in Te Reo Māori, Tongan, Samoan, Korean, and Simplified Chinese). They will be invited to: 1) attend the clinic, give informed consent, and take the sample (e.g. in the clinic bathroom); 2) return the kit to the practice nurse; and 3) complete a questionnaire on: i) their experience; ii) the acceptability of the self-sampling process; and iii) their preferences.

The mail-out self-sampling group will receive an invitation letter and the self-sampling kit and information package in the mail. The self-sampling kit and information package will contain: 1) the participant information brochure; 2) an informed consent document; 3) a laboratory request form; 4) a questionnaire regarding acceptability; 5) a swab and tube; 6) instructions on how to take the sample and to return it as soon as possible; and 7) a return, free, pre-addressed courier bag. They will also be directed to the webpage with translated study documents and the study video clips.

Once samples are collected, the tubes will be placed in a biohazard bag together with the consent/laboratory test request form, and placed in the courier pick-up area for samples taken at a clinic or into the courier bag for samples taken at home. Women may also choose to drop their sample back at their general practice clinic or direct to the laboratory. Samples will be stored and transported at room temperature. The number of days between collection of the sample and receipt by the laboratory will be recorded.

Four weeks after the invitation letters have been sent, non-responding women will be sent a reminder text message.

#### Opportunistic sub-study invitation

In a subset of general practices, study subjects who have not participated in the study 3 months after invitation will have an alert placed on the clinic practice-management system stating that they are eligible for the sub-study. They will also be sent a letter or text informing them that they are now able to self-sample at their clinic or request that a self-sampling kit be mailed to them, both for a limited period of time. At any clinic-visit over the subsequent 6 months, the alert will notify the clinic to offer self-sampling and the study subjects will be able to take the test at the clinic and complete the questionnaire. Clinic staff will be trained to consent women to the study and to manage results. The same outcomes will be measured in the sub-study as in the main study.

#### Study materials

With permission from authors of the iPAP trial, iPAP materials for women have been adapted to local language usage and cultural context (‘localised’) and redeveloped into a participant information brochure, consent materials, results information and laboratory forms as part of the New Zealand feasibility study [43]. The localisation included ensuring that findings from qualitative studies for iPAP [44] (e.g. addressing women’s concerns of not collecting the sample correctly and highlighting that HPV testing is not a test of spouse/partner fidelity) were addressed.

#### Questionnaire

Based on the Australian iPap study-responders post-test questionnaire [45], a questionnaire regarding acceptability has also been developed and localised. The questionnaire includes information on the women’s experience with the self-sampling procedure (including pain, discomfort, and privacy or embarrassment concerns); aspects of feasibility (such as ease of use, confidence in the test, and confidence of correctly taking the sample themselves compared with confidence of a health-professional correctly taking the sample); practical issues (such as ease of getting an appointment with a smear-taker); the women’s preference for specimen-collection technique (self-sampling or a clinician-collected sample); their willingness to participate in self-sampling-based screening in the future; whether they viewed the study videos; and demographic details (such as level of education and socioeconomic position). The questionnaire also asks whether the instructions for the self-sampling technique were clear, to enable us to ensure that any instructions in the future are clear and acceptable to Māori, Pacific, and Asian women.

A separate, short questionnaire has been developed – and will be administered, with verbal consent, by telephone – to a random sample of approximately 24 non-responding women to examine their reasons for non-participation.

### Laboratory testing

#### Cytology testing

Cytology testing will be carried out in accordance with NCSP standards [46].

#### HPV testing

All samples will be tested for the presence of oncogenic HPV DNA using the clinically validated cobas 4800 HPV assay [29, 47] (Roche Molecular Systems, Pleasanton, California, USA) at a single accredited laboratory. The cobas 4800 HPV assay is approved by the US FDA for primary HPV screening (other HPV DNA assays are approved for use in conjunction with cytology) [48]; fulfils the New Zealand-specific criteria for HPV testing [46]; and has been selected by the Netherlands for primary HPV testing [49]. This assay specifically detects HPV types 16 and 18, as well as 12 other oncogenic HPV types as a group [48]. The protocol for testing self-taken swabs on the cobas HPV test is not validated by the manufacturer (Roche), but was validated by an Australian study [31]. On receipt in the laboratory, samples will be irrigated in 4 mL of PreservCyt buffer, and vortexed for at least 30 s prior to decapping and loading in swab-sample carriers on the cobas 4800. The cobas HPV test is then run according to the manufacturer’s instructions. All failed and invalid samples will be recorded and a repeat sample requested.

#### Future laboratory analyses

With women’s consent, we will freeze an aliquot of each self-test sample that had a positive test result for oncogenic HPV for future HPV ‘other’ type differentiation. No analysis of human tissue or human genetic analysis will be performed; the only potential future analysis will be of specific viral DNA related to understanding the prevalence of non16/18 types of oncogenic HPV’s in the study population.

### Results management

#### Negative results management

Negative results (i.e., samples showing no evidence of HPV) will be provided to women by letter, text message, or telephone call by the usual primary-care provider and they will be advised to return for a routine cervical screen at the appropriate clinical interval, as specified by the NCSP guidelines and an amended approach to the proposed HPV primary-screening algorithm, with agreement from the NCSP (3 year recall interval) [37].

#### Cytology results management

Women with inadequate or abnormal cytology (taken in usual care or as follow-up to a HPV ‘other’ result) will be followed-up according to the NCSP guidelines [37] by the requesting smear-taker.

#### Oncogenic HPV results management

Positive HPV results will be managed as per the current NCSP guidelines, with adjustments in accordance with NCSP clinical advice [50]. The majority of women will be informed of their test results over the phone, with a few being informed in person by their usual primary-care provider. Women who test HPV16/18 positive will be referred directly to colposcopy. In order to avoid an additional cytology test (since this is potentially a barrier to follow-up), ‘blind’ colposcopy will be conducted (i.e. colposcopy will be performed before cytology, with the cytology sample being taken at colposcopy). Women who test positive for the pool of 12 other oncogenic HPV types will be triaged (at no cost to the woman) with a clinician-conducted cervical-cytology test (i.e. the current standard screening test); however, if the woman declines cytology, she may be offered a colposcopy to ensure safe follow-up. In accordance with NCSP guidelines, [50] women who return cell changes greater than low-grade on cytology will also be referred to colposcopy and women whose cytology is low-grade or less will be referred for management by their usual primary-care provider team for a repeat cytology test after 1 year.

#### Clinical follow-up

To ensure clinical safety, the return-of-results primary-care clinician will have 10 days to contact the woman, during which they will explain the results and, in the case of HPV16/18-positive women, make a referral for colposcopy. In the case of women who have a positive test result for other oncogenic HPV types, the return-of-results clinician will make an appointment to take a cytology sample. The study nurse will monitor positive results and provide a failsafe follow-up process: if the participant is not informed in the 10-day timeframe, the study research nurse will work with the GP and provide, or refer for, support-to-service to ensure that women are notified and offered support to attend appropriate follow-up or provide the follow-up themselves. Non-attenders for abnormal cytology results will be followed up until the end of the study by the study nurse and then by their usual primary-care providers.

All screening elements will be free to participants. We will partner with local primary-care, support-to-service providers, Māori/Pacific/Asian Providers, and DHB colposcopy services to design and test appropriate support-to-service strategies to ensure that the majority of women who test positive for oncogenic HPV will be seen in clinic. This support will be tailored to meet women’s needs and may include transport, childcare, and visit-attendance support.

#### Collection of clinical results

We will obtain the results of any subsequent cervical cytology, colposcopy, and histology from the NCSP-Register or GPs, ISPs, Māori/Pacific/Asian Providers, and DHB colposcopy services in order to determine prevalence of cervical abnormalities.

### Data entry, management and security

A bespoke secure online database has been created to manage details of responding and non-responding participants. Access databases will be developed for data entry from the study questionnaires. Any specimen taken for screening as part of the study is reported to the NCSP-Register by direct laboratory notification.

An internal data safety monitoring committee has been established (public-health physician; molecular HPV expert; general practitioner; colposcopist; nurse smear-taker; research nurse; epidemiologist) to assess and evaluate any clinical concerns or serious adverse events occurring in the study.

### Statistical analysis

The main analyses will focus on the primary outcome of the study: participation, i.e. the proportion of women who provide a self-sample compared with the proportion who attend for cytology, stratified by ethnicity. These will initially be assessed simply by comparing (with Chi-square tests) the proportions who participated in each group; this will be followed by a mixed model multiple logistic regression analysis to adjust for potential confounders (e.g. age, ethnicity, screening history, socioeconomic status) and to assess which factors (in addition to the intervention) affect participation. The same analyses will be repeated separately for both un- and under-screened women, providing important information on whether screening history affects participation.

We will also analyse the prevalence of oncogenic HPV types in participating women in the self-sampling groups, and the prevalence of cervical abnormalities in participating women in the cytology group. Prevalence odds ratios [51] will be calculated using logistic regression and compared across ethnicities, adjusting for age.

Secondary analyses will assess the association between acceptance of self-sampling and demographic and other factors. For comparisons across ethnicity and screening-history (un- and under-screened) groups, logistic regression will be used, adjusting for age and other potential confounding variables. Time for return of sample will be calculated for self-sampling groups as a policy-relevant indicator of courier reliability; laboratory turnaround time and proportion of unsatisfactory samples will be monitored.

Experience of the test, enablers/barriers of screening, and factors associated with screening preferences will be described for each group (mail-out or clinic-based), by ethnicity and demographic factors. Standard descriptive statistical methods will be used. T-tests and Chi-square tests will be used to assess statistical significance. Multiple linear or logistic regression (as appropriate) will be used to compare these responses between the two groups (mail-out or clinic-based), adjusting for potential confounders.

### Sample size

We aim to invite a minimum of 3550 un- or under-screened (≥5 years overdue) Māori, Pacific and Asian women (1050, 1250, 1250 respectively) for screening. The invitation numbers are pragmatically based on the number of eligible women in the region and study resources. With 450 Māori women invited in both the clinic and mail-out self-sampling groups we will have > 95% power to detect a 10% difference in uptake between the groups (e.g.*,* 15% uptake in the clinic group and 25% in the mail-out group). With 450 Māori women invited in the clinic self-sampling group and 150 Māori women invited in the usual care group we will have > 85% power to detect a 10% difference in uptake between the groups (e.g.*,* 15% uptake in the clinic group and 5% in the usual care group).

### Ethical and cultural considerations

The study has been approved by the New Zealand Northern B Health and Disability Ethics Committee (HDEC) (reference: 17/NTB/120). The New Zealand Ministry of Health (including the National Kaitiaki Group) and the participating DHBs, PHOs, and primary-care clinics have all given approval for the use of data to identify and contact eligible women.

There are a range of ethical and cultural issues in this study. The issues include informed consent, privacy and confidentially, language barriers (especially for Pacific and Asian women), health literacy and awareness of screening, sampling and storage of tissue, data ownership, and that for some women (especially Māori women) the genital area is considered tapu (sacred/forbidden/taboo). Whakamā (embarrassment/reticence/shyness) may be an issue for some women in this study, and appropriate ways to approach women will be co-designed with each provider; a range of strategies are likely to be required. There is also the issue of potential stigmatisation (e.g. a deficit focus for un-screened/under-screened women); however, this study seeks a strength-based approach of enabling women to access a novel technology to enhance their wellbeing. Our research group includes substantial research expertise with Māori health, Māori research methodology, and research with women, screening, and in cervical screening specifically. The study has an advisory group structure for each of the study populations: Māori, Pacific and Asian.

## Discussion

The success of cervical-cancer screening in New Zealand is limited by incomplete participation, particularly amongst Māori, Pacific and Asian women. More than half of the invasive cervical cancer cases among these populations occur in those who have not attended cervical screening [5]. Improving participation rates and reducing outcome inequities are priorities for New Zealand’s health system. The longstanding nature of these problems shows that new strategies are needed. HPV self-sampling may improve the participation rates in groups of women who are underserved by current screening programmes that require a clinician-collected sample [34]. However, it is important to assess the acceptability and optimal invitation approaches for self-sampling in New Zealand in order to provide the country-specific evidence needed to inform policy. The current trial will provide robust evidence on whether HPV self-sampling could be used to increase cervical screening participation rates in un- and under-screened Māori, Pacific, and Asian women. This trial is the first to evaluate the effectiveness of mailed self-sampling for cervical-cancer screening in New Zealand, and one of the first internationally to evaluate the effectiveness of opportunistic in-clinic invitations for self-sampling.

## Data Availability

Anonymized data and biological samples will be collected during the trial and used only for the analysis declared in the study protocol. The availability of the data that will be collected as part of this study is being confirmed.

## Abbreviations

ANZCTR: Australian and New Zealand Clinical Trials Registry
ASC-H: Atypical squamous cells - cannot exclude high-grade squamous intra-epithelial lesion
ASC-US: Atypical squamous cells of undetermined significance
CIN2 +: Cervical intraepithelial neoplasia grade two or higher
CIN3+: Cervical intraepithelial neoplasia grade three or higher
DHB: District Health Board
DNA: Deoxyribonucleic acid
GP: General Practice
HDEC: Health and Disability Ethics Committee
HPV: Human papillomavirus
HSIL: High-grade squamous intra-epithelial lesion
ISP: Independent Service Providers
LBC: Liquid-based cytology
LSIL: Low-grade squamous intra-epithelial lesion
NCSP: New Zealand National Cervical Screening Programme
PCR: Polymerase chain reaction
PHO: Primary Health Organisation
US FDA: United States Food and Drug Administration
