# Correction to: Comparison of two invitation-based methods for human papillomavirus (HPV) self-sampling with usual care among un- and under-screened Māori, Pacific and Asian women: study protocol for a randomised controlled community trial to examine the effect of self-sampling on participation in cervical-cancer screening

**DOI:** 10.1186/s12885-020-6671-4

**Published:** 2020-02-27

**Authors:** Naomi Brewer, Karen Bartholomew, Anna Maxwell, Jane Grant, Georgina McPherson, Helen Wihongi, Collette Bromhead, Nina Scott, Sue Crengle, Chris Cunningham, Jeroen Douwes, John D. Potter

**Affiliations:** 1 Centre for Public Health Research, College of Health, Massey University, PO Box 756, Wellington, 6140 New Zealand; 2 Waitematā District Health Board (DHB) and Auckland DHB, Private Bag 93-503, Takapuna, Auckland, 0740 New Zealand; 3 Waitematā District Health Board, Auckland, New Zealand; 4 School of Health Sciences, Massey University, Wellington, New Zealand; 5 University of Auckland, Waikato District Health Board, Hamilton, New Zealand; 6 Department of Preventive and Social Medicine, University of Otago, Dunedin, New Zealand; 7 Research Centre for Māori Health and Development, Massey University, Wellington, New Zealand; 8 Centre for Public Health Research, Massey University, Wellington, New Zealand

**Correction to: BMC Cancer**

**https://doi.org/10.1186/s12885-019-6401-y**

Following publication of the original article [1], the authors reported an error in the authorship list associated with the paper. Georgina McPherson has therefore been added.
